# Histone demethylase PHF8 drives neuroendocrine prostate cancer progression by epigenetically upregulating FOXA2

**DOI:** 10.1002/path.5557

**Published:** 2020-11-05

**Authors:** Qiuli Liu, Jian Pang, Lin‐ang Wang, Zhuowei Huang, Jing Xu, Xingxia Yang, Qiubo Xie, Yiqiang Huang, Tang Tang, Dali Tong, Gaolei Liu, Luofu Wang, Dianzheng Zhang, Qiang Ma, Hualiang Xiao, Weihua Lan, Jun Qin, Jun Jiang

**Affiliations:** ^1^ Department of Urology, Institute of Surgery Research Daping Hospital, Army Medical University Chongqing PR China; ^2^ Department of Bio‐Medical Sciences Philadelphia College of Osteopathic Medicine Philadelphia PA USA; ^3^ Department of Pathology, Institute of Surgery Research Daping Hospital, Army Medical University Chongqing PR China; ^4^ CAS Key Laboratory of Tissue Microenvironment and Tumor, CAS Center for Excellence in Molecular Cell Science, Shanghai Institute of Nutrition and Health Sciences Chinese Academy of Sciences, University of Chinese Academy of Sciences Shanghai PR China

**Keywords:** prostate cancer, neuroendocrine, PHF8, FOXA2, histone, demethylase, epigenetic, transcriptional factor, TRAMP mice, tissue microarray

## Abstract

Neuroendocrine prostate cancer (NEPC) is a more aggressive subtype of castration‐resistant prostate cancer (CRPC). Although it is well established that PHF8 can enhance prostate cancer cell proliferation, whether PHF8 is involved in prostate cancer initiation and progression is relatively unclear. By comparing the transgenic adenocarcinoma of the mouse prostate (TRAMP) mice with or without *Phf8* knockout, we systemically examined the role of PHF8 in prostate cancer development. We found that PHF8 plays a minimum role in initiation and progression of adenocarcinoma. However, PHF8 is essential for NEPC because not only is PHF8 highly expressed in NEPC but also animals without *Phf8* failed to develop NEPC. Mechanistically, PHF8 transcriptionally upregulates FOXA2 by demethylating and removing the repressive histone markers on the promoter region of the *FOXA2* gene, and the upregulated FOXA2 subsequently regulates the expression of genes involved in NEPC development. Since both PHF8 and FOXA2 are highly expressed in NEPC tissues from patients or patient‐derived xenografts, the levels of PHF8 and FOXA2 can either individually or in combination serve as NEPC biomarkers and targeting either PHF8 or FOXA2 could be potential therapeutic strategies for NEPC treatment. © 2020 The Authors. *The Journal of Pathology* published by John Wiley & Sons, Ltd. on behalf of The Pathological Society of Great Britain and Ireland.

## Introduction

Neuroendocrine prostate cancer (NEPC) is a more aggressive subtype of castration‐resistant prostate cancer (CRPC) and therefore patients with NEPC have an extremely poor overall survival [1]. Although only a small percentage (0.5–2%) of primary prostate cancers are NEPC [2], treatments such as androgen deprivation therapy (ADT), especially with the use of the second‐generation anti‐androgen drugs enzalutamide and abiraterone, often induce neuroendocrine differentiation and lead to up to 30% of metastatic castration‐resistant tumors as NEPC [3]. Compared with the other subtypes of prostate cancer, NEPC expresses specific neuroendocrine markers including synaptophysin (SYP), chromogranin A (CgA), and neuronal‐specific enolase (NSE), with concurrent absence of androgen receptor (AR) and prostate‐specific antigen (PSA) [4]. Due to the limited understanding of the molecular mechanisms in NEPC development and progression, there are few options for NEPC treatment except platinum‐based chemotherapies.

Although the origin of NEPC cells remains debated, different molecular mechanisms might be involved in NEPC development. Previous studies suggest that NEPC may originate from neuroendocrine cells [5], cancer stem cells [6], p63‐positive basal cells [7] or CRPC adenocarcinoma cells via trans‐differentiation [8, 9], especially under the pressure of ADT [10, 11]. There is compelling evidence that loss of tumor suppressors such as RB1 and p53 is required to develop a neuroendocrine lineage [12]. In addition, several transcription factors including MYCN [13], BRN2 [14], FOXA2 [15], and ASCL1 [16], as well as epigenetic regulators such as REST [17] and EZH2 [18], have been implicated in NEPC development. Of note, FOXA2, a member of the FOXA family of the Forkhead box transcription factors, has been implicated as a specific marker for small cell NEPC [19, 20]. Furthermore, results from research using the transgenic adenocarcinoma of the mouse prostate (TRAMP) mouse model indicate that FOXA2 functions together with HIF1α to drive a transcriptional program essential for NEPC development [15], thus establishing FOXA2 as a driving factor for NEPC.

PHF8 (plant homeodomain finger‐containing protein 8), also known as KDM7B, is a histone demethylase. The N‐terminal plant homeodomain (PHD) domain of PHF8 is responsible for recognizing/binding di‐ and tri‐methylated histone H3 lysine 4 (H3K4me2/3), which are often enriched at transcription start sites, whereas its C‐terminal JmjC domain possesses histone demethylase activities, catalyzing the removal of methyl group from H3K9me1/2, H3K27me2, and H4K20me1 [21]. PHF8 has been implicated in promoting different malignancies including prostate cancer [22], esophagus cancer [23], and lung cancer [24]. We previously used a prostate cancer cell line‐based model to demonstrate that PHF8, by serving as an AR coactivator, can enhance CRPC progression [25]. In this study, we used the well‐established TRAMP mouse model with *Phf8* knockout to demonstrate the critical role of PHF8 and the underlying molecular mechanism in PHF8‐mediated NEPC development.

## Materials and methods

### Animals and experimental procedures

All experiments involving animals were performed in accordance with international laws (EEC Council Directive 86/609, OJ L 358. 1, 12 December 1987; Guide for the Care and Use of Laboratory Animals, United States National Research Council, 1996) and approved by the Institutional Animal Care and Use Committee of Army Medical University. Transgenic TRAMP (transgenic adenocarcinoma of the mouse prostate, C57BL/6) and *Phf8* knockout mice at the age of 5–6 weeks were obtained from the Model Animal Research Center of Nanjing University (Nanjing, Jiangsu, PR China). The animals were housed and crossed in the Experimental Animal Center of Daping Hospital, Army Medical University (Chongqing, PR China). By crossing *Phf8* knockout female mice (*Phf8* ^X−/−^) with TRAMP male mice, *Phf8* knockout TRAMP mice (*Phf8*‐KO; TRAMP) were obtained. Mice were sacrificed at weeks 12 (*n* = 5 per group), 25 (*n* = 5 per group), 37 (*n* = 8 per group), and 42 (*n* = 7 per group).

### Histologic analysis

The prostate was dissected from sacrificed animals and fixed in 10% formalin for 24 h, then paraffin‐embedded for hematoxylin and eosin (H&E) staining. Histologic analysis of prostate lesions including NEPC was conducted essentially as described previously [26]. In brief, the slides were observed using a light microscope (BX53; Olympus, Tokyo, Japan), photographed, and the histologic features were classified by two certified pathologists double‐blindly (QM and HX) based on the following specifications: (1) normal tissue (NT); (2) low‐grade PIN (prostatic intraepithelial neoplasia, LGPIN); (3) high‐grade PIN (HGPIN); (4) well‐differentiated adenocarcinoma (WD‐Adeno); and (5) undifferentiated adenocarcinoma (UD‐Adeno, which was categorized as an NEPC lesion according to the pathological characteristics: pleomorphism, high nuclear/cytoplasmic ratio, and loss of glandular differentiation). For each prostate, ten random fields were captured at 10× magnification, which were further divided into four quadrants. In each quadrant, the most advanced histologic feature was used for histological classification. Thus, the numbers of each subtype of lesions in each experimental group were established, and the percentages of different subtypes of lesions were compared among different experimental groups.

### Cell lines, reagents, and patient‐derived xenograft (PDX) tissue lines

The prostate cancer cell lines PC‐3 and LNCaP were obtained from Cell Bank of Shanghai Institutes for Biological Sciences (Chinese Academy of Sciences, Shanghai, PR China), and NE1.3 cells were kindly provided by Dr Wenliang Li (University of Texas Health Science Center at Houston; MD Anderson Cancer Center UT Health Graduate School of Biomedical Sciences). All cells were grown in the medium recommended by the distributors. PDX tissue lines (LTL‐545 and LTL‐313HR) were kindly provided by Professor Yuzhuo Wang (Living Tumor Laboratory, http://livingtumorlab.com/). The specimens were transplanted subcutaneously into NPG mice (Beijing Vitalstar Biotechnology, Beijing, PR China). The transplanted tumors were dissected from the sacrificed mice, fixed in 10% formalin, and paraffin‐embedded for immunostaining and examination.

### siRNA transfections and shRNA infections

siRNAs (Guangzhou RiboBio Co, Ltd, Guangzhou, Guangdong Province, PR China) were used to transiently knock down PHF8 in cells with scrambled siRNA as the controls. The specific siRNAs against *PHF8* were as follows: si*PHF8*‐1 (CCGGAGACAGTGCGAACCGTA and CAGCCTTAACATCGAGATGCA); si*PHF8*‐2 (TCGGCGAACCAAGATAGCAAA and CAGGTGATGGAAGACGAATTT). In general, siRNAs (150 nm) were transfected into 5 million cells using Lipo2000 according to the manufacturer's protocol (Thermo Fisher, Waltham, MA, USA) and the cells were analyzed 48 h post‐transfection. To increase knockdown efficiency, four respective specific siRNAs were pooled for transfection. For the stable knockdown by using shRNA infections, *PHF8* shRNA (targeted sequence: GCTCTTTCCAGAAAGCAAAGT) was cloned into pYr‐LVsh‐EGFP‐Puro vector (Changsha Yingrun Biotechnology Co, Ltd, Changsha, Hunan Province, PR China). PC‐3, LNCaP, and NE1.3 cells were infected with EV (empty vector as control) or *PHF8* shRNA lentivirus, and puromycin was used to select the infected cells.

### *In vivo* tumorigenesis assay

PC‐3 cells (1 × 10^7^ per 50 μl) with (sh*PHF8*) or without (EV) infection of *PHF8* shRNA were mixed with Matrigel (1:1) and then injected subcutaneously into the flanks of 6‐ to 7‐week‐old nude mice. The sizes of the tumors were recorded every 2 days and the volumes were calculated using the formula length × width^2^/2. The final tumor size was estimated using the whole‐body fluorescence as the pYr‐LVsh‐Puro vector contained the EGFP sequence and the xenograft tumors derived from EV and sh*PHF8* PC‐3 cells could be detected using a whole‐body fluorescence imaging system (Maestro in vivo Imaging System; PerkinElmer, Waltham, MA, USA). The transplanted tumors were dissected, weighed, fixed in 10% formalin, and paraffin‐embedded for immunostaining examination. To assess any difference in lung metastasis, EV and sh*PHF8* PC‐3 cells (3 × 10^6^ per 50 μl) were injected into the tail vein of 6‐ to 7‐week‐old nude mice and the tumors were allowed to grow for 19 days before mice were sacrificed. The lungs were dissected, rinsed with PBS, and paraffin‐embedded for sectioning.

### Patients and tissue samples

All procedures involving human participants were carried out in accordance with the ethical standards of the institutional research committee and with the 1964 Declaration of Helsinki and its later amendments or comparable ethical standards. All patient samples were collected by the Department of Pathology with approval from the Research Ethics Committee of Daping Hospital, Army Medical University, and written informed consent was obtained from each patient. To estimate the role of PHF8 in ADT‐induced NED, a cohort of seven patients was specifically selected from our database. All these patients underwent ADT and abiraterone, enzalutamide or docetaxel treatment after prostate puncture biopsy, and NEPC or NE regions were identified after the therapy. The clinical information of these patients is shown in supplementary material, Table S1. To determine the levels of PHF8 and FOXA2 in adenocarcinoma, CRPC‐Adeno, and NEPC tissues, an adenocarcinoma tissue microarray (TMA) described previously [27], a PDX tissue microarray kindly provided by Professor Yuzhuo Wang, as well as another six CRPC‐Adeno tissues (supplementary material, Table S2) and the above‐mentioned seven NEPC or NED tissues from our cohort were used. In total, 59 samples with adenocarcinoma, 13 samples with CRPC‐Adeno, and 10 samples with NEPC or NED were included.

### Immunohistochemical staining

The embedded specimens were sectioned, mounted onto glass slides, and then immunostained as described previously [27]. Primary antibodies against AR (ab108347, 1:200; Abcam, Cambridge, MA, USA), PSA (sc‐7316, 1:50; Santa Cruz Biotechnology, Dallas, TX, USA), SYP (17785‐1‐AP, 1:400; Proteintech, Rosemont, IL, USA), CD56 (14255‐1‐AP, 1:600; Proteintech), CgA (60135‐1‐lg, 1:600; Proteintech), PHF8 (ab36068, 1:200; Abcam), Large‐T (554149, 1:100; BD, Lake Franklin, NJ, USA), and FOXA2 (sc374376, 1:50; Santa Cruz Biotechnology) were used. The staining intensities were scored as described previously [28] by certified pathologists (QM and HX) and imaged using a light microscope (BX53; Olympus, Tokyo, Japan).

### Immunofluorescence staining

Immunofluorescence staining for CK5 and CK18 or PHF8 and FOXA2 was carried out on formalin‐fixed, paraffin‐embedded sections. The sections were incubated with primary antibodies against CK5 (ab52635, rabbit, Abcam, 1:400), CK18 (66187‐1‐lg, mouse, Proteintech, 1:200), PHF8 (ab36068, rabbit, Abcam, 1:200), and FOXA2 (sc374376, mouse, Santa Cruz Biotechnology, 1:50). Secondary antibodies used for detection were goat anti‐rabbit antibodies conjugated to Alexa Fluor 488 (1:200; Zhongshan Gold Bridge Biotechnology, Beijing, PR China) and goat anti‐mouse antibodies conjugated to Alexa Fluor 594 (1:200; Zhongshan Gold Bridge Biotechnology). The sections were counterstained with DAPI for 15 min (KGA215; KeyGenBioTECH, Nanjing, Jiangsu Province, PR China) at 37 °C, and images were obtained using confocal microscopy (LSM700; Zeiss, Oberkochen, Germany).

### Estimation of cell number

PC‐3, LNCaP, and NE1.3 cells with or without infection with *PHF8* shRNA and/or *FOXA2* plasmid were seeded into 96‐well plates. Cell numbers were evaluated using the Cell Counting Kit‐8 (CCK‐8) assay at the indicated time points. In brief, the culture medium was aspirated and the cells were rinsed with PBS, followed by application of CCK‐8 reagent (10 μl per well) for 2 h at 37 °C. Absorbance at 450 nm was measured spectrophotometrically (Bio‐Rad, Hercules, CA, USA).

### Clonogenic assays

PC‐3 cells with or without infection of *PHF8* shRNA and LNCaP cells with or without PHF8 overexpression were seeded in six‐well plates with 1000 (PC‐3) or 2000 (LNCaP) cells per well, treated as indicated, and incubated at 37 °C for 14 days with the medium changed every 7 days. At the end of the experiment, cells were fixed with methanol, stained with crystal violet, and the numbers of colonies were counted.

### Transwell assays

To assess cell migration and invasion *in vitro*, we used 24‐well Transwell chambers with or without Matrigel. PC‐3 and LNCaP cells with or without *PHF8* shRNA were trypsinized and seeded into the top chamber at a density of 5 × 10^4^ cells per well in 200 μl of Dulbecco's modified Eagle's medium. The bottom chambers contained 800 μl of medium (10% fetal calf serum). After incubation at 37 °C for 24 h (PC‐3) or 48 h (LNCaP), cells attached to the top of the membrane were carefully removed with a cotton swab, whereas cells in the bottom chamber were fixed with 10% formalin and stained with crystal violet for 3 min at room temperature and counted.

### Wound healing assay

PC‐3 cells with or without shRNA against *PHF8* were seeded into a six‐well plate and grown for 24 h. A scratch was made in the confluent cell monolayer using a sterile 10‐μl pipette tip and the cells were rinsed once with PBS and then incubated in a serum‐free medium. Images of the scratches were captured at different time points (0, 24, and 48 h) with an inverted microscope (CK40F200; Olympus).

### Luciferase assay

Cells in 12‐well plates were transfected with 1 μg of *FOXA2*‐Luc vector, 100 ng of *Renilla* luciferase vector, and plasmid expressing *PHF8*, using Lipofectamine. Transfected cells were cultured for 48 h and the cell lysates were used for a Dual‐luciferase Assay (E1910; Promega, Madison, WI, USA). Ten microliters of cell lysates was loaded into a 96‐well plate and luciferase activity was measured using a Veritas Microplate Luminometer (Thermo Fisher, Waltham, MA, USA) according to the manufacturer's instructions. *Renilla* luciferase activity was used to normalize transfection efficiency.

### Immunoblotting

Cell lysates were separated by SDS‐PAGE, followed by immunoblotting assays as described previously [27, 28] with antibodies against PHF8 (ab36068, 1:1000; Abcam, Cambridge, MA, USA), SYP (17785‐1‐AP, 1:2000; Proteintech, Rosemont, IL, USA), CgA (60135‐1‐lg, 1:600; Proteintech), CD56 (14255‐1‐AP, 1:2000; Proteintech), FOXA2 (ab256493, 1:1000; Abcam), and β‐actin (3700s, 1:5000; Cell Signaling Technology, Boston, MA, USA).

### Chromatin immunoprecipitation (ChIP) assays and quantitative polymerase chain reaction (qPCR)

ChIP assays were performed using a magnetic ChIP kit according to the manufacturer's instructions (53009; Active Motif, Carlsbad, CA, USA). In brief, cells were fixed by 1% formaldehyde; chromatins were fragmented by enzymatic digestion. Antibodies against PHF8 (ab36068; Abcam), H3K9me1 (ab8896; Abcam), H3K9me2 (ab1220; Abcam), H3K27me2 (ab24684; Abcam), and H4K20me1 (ab9051; Abcam) were used for immunoprecipitation. After washing and reverse‐crosslinking, the precipitated DNA was purified and amplified by qPCR. Extracted RNA was reverse‐transcribed into cDNA and qPCR was performed using the SYBR Green Master Mix (Invitrogen, Carlsbad, CA, USA) in the iCycle System (Bio‐Rad, Hercules, CA, USA). PCR data were analyzed using GraphPad Prism (GraphPad Software, San Diego, CA, USA). The sequences of the PCR primers used are listed in supplementary material, Table S3.

### RNA‐seq analysis

Total RNA was extracted from tumor tissues derived from either *Phf8*‐KO TRAMP mice or the control TRAMP mice at week 37 using Trizol reagent (Invitrogen). RNA‐seq was performed by Shanghai NovelBio Bio‐Pharm Technology Co, Ltd (Shanghai, PR China). Each sample contained pooled RNA from three mice in each group and was mixed with an equal mass of RNA to minimize variation across samples. RNA‐seq reads were filtered and mapped to the mouse genome (GRCm38, NCBI) utilizing HISAT2 [29]. HTSeq was used to calculate the gene count [30]. Differentially‐expressed gene analysis was applied utilizing DESeq2 [31] under the following criteria: fold‐change > 2, or fold‐change < 0.5; FDR < 0.05. Gene set enrichment analysis (GSEA) was performed on the data from the Hallmark pathway database [32]. The raw data can be downloaded from GEO GSE157621 (https://www.ncbi.nlm.nih.gov/geo/query/acc.cgi?acc=GSE157621).

### Statistical analysis

SPSS 19.0 (IBM, Amonk, NY, USA) was used for all statistical analyses. Data are presented as mean ± SD. If the data followed a normal distribution, the statistical significance of differences between two groups of data was analyzed by a *t*‐test or paired *t*‐test or χ^2^ test, and differences among several groups were analyzed by one‐way analysis of variance followed by the least significant difference procedure for comparison of means. For data that did not fit a normal distribution, nonparametric statistical tests were used. Kendall rank correlation analysis was used for the analysis of the correlation of the expression of PHF8 and FOXA2. A *P* value of less than 0.05 was considered statistically significant.

## Results

### PHF8 plays an important role in NEPC development without affecting tumor initiation

The *Phf8* knockout male mice (*Phf8* ^−/y^ mice) were generated as described previously [33]. Consistent with the previous observation, loss of *Phf8* had no effect on either gross morphological phenotype or fertility (data not shown). In addition, histopathological staining of prostates from 25‐, 37‐, and 53‐week‐old control C57 and *Phf8* ^−/y^ male mice showed that *Phf8* knockout did not affect prostate development, suggesting that PHF8 is not essential for prostate homeostasis under physiological conditions (supplementary material, Figure S1A–C). This observation was further substantiated by double immunofluorescence staining for CK5 (a marker of basal cells) and CK18 (a marker of luminal epithelial cells) (supplementary material, Figure S1D–F).

**Figure 1:**
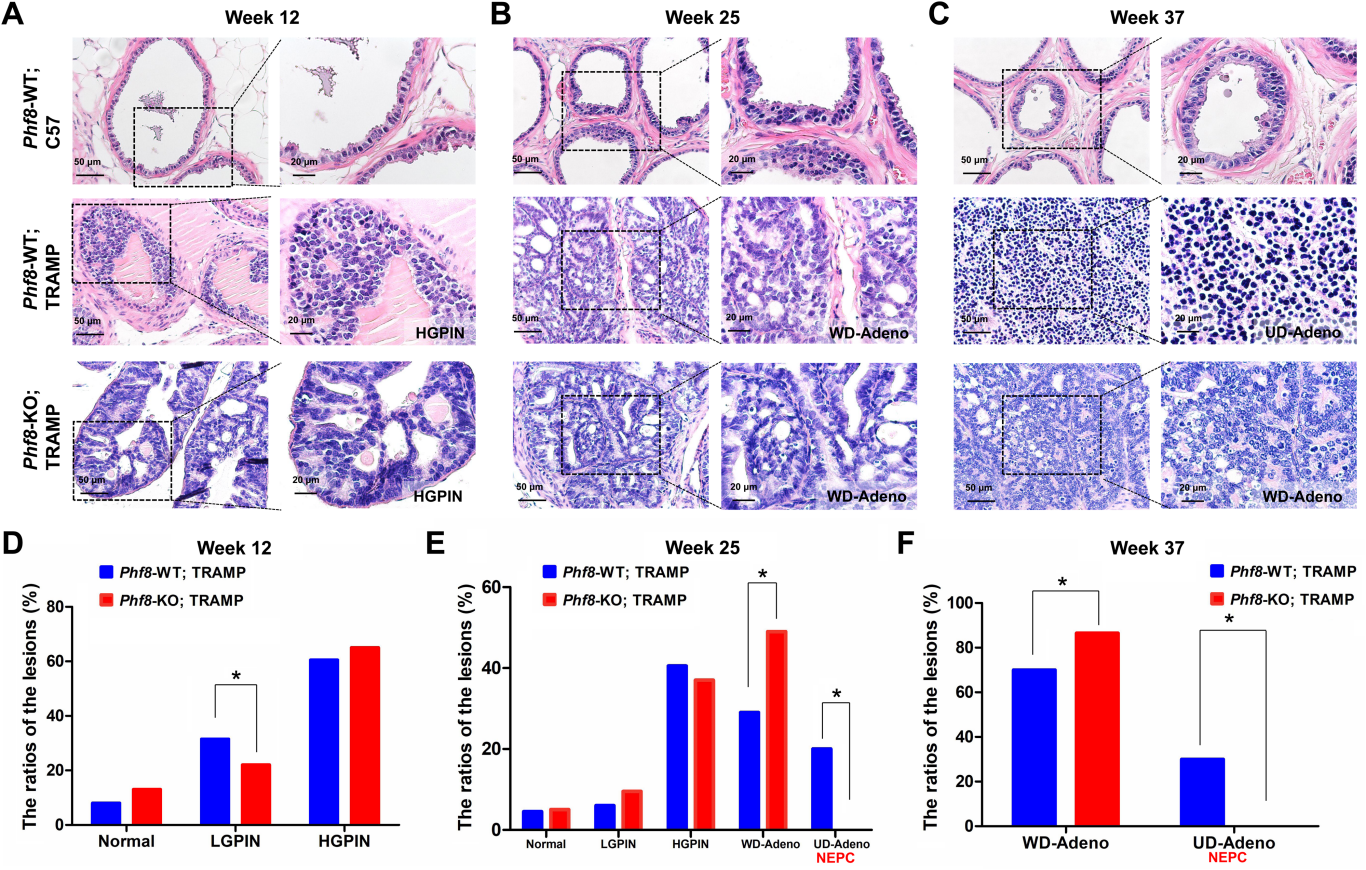
Tumorigenesis in *Phf8*‐KO TRAMP mice. (A–C) The predominant histologic classifications of the prostatic lesions of the mice at weeks 12 (A), 25 (B), and 37 (C). (D–F) The statistical results (χ^2^ test) of the histologic analysis between *Phf8*‐WT and *Phf8*‐KO TRAMP mice at weeks 12(D), 25 (E), and 37 (F).

The TRAMP mouse model has been widely used in prostate cancer research because it closely mirrors the pathogenesis of human prostate cancer. With the transgenic expression of viral SV40 oncoprotein in the prostatic epithelium, TRAMP mice develop PIN at 10–12 weeks of age and invasive prostate adenocarcinoma by 18–20 weeks [34, 35]. In their lifetime, about 20% of TRAMP mice develop NEPC [5]. To investigate the potential role of PHF8 in prostate cancer initiation and progression, we crossed female *Phf8* knockout mice (*Phf8* ^X−/−^) with male TRAMP mice to obtain male *Phf8* knockout TRAMP mice (TRAMP/*Phf8*‐KO). We then compared prostate lesions in TRAMP/*Phf8*‐KO male mice with those of TRAMP male mice at weeks 12, 25, and 37. Prostate lesions were categorized as normal, LGPIN or HGPIN, WD‐Adeno or UD‐Adeno, and analyzed as described previously [36]. Also according to a previous study [26], we categorized UD‐Adeno as NEPC lesions, because our H&E staining revealed that the cells in UD‐Adeno lesions show NEPC‐like characteristics including pleomorphism, a high nuclear/cytoplasmic ratio, and loss of glandular differentiation. Figure 1A–C show representative H&E stains of the prostate lesions from control TRAMP and TRAMP/*Phf8*‐KO male mice at weeks 12, 25, and 37, respectively, and Figure 1D–F show the corresponding lesion status at each stage. Both LGPIN and HGPIN were observed in both TRAMP/*Phf8*‐WT and TRAMP/*Phf8*‐KO mice (*n* = 5) at week 12. Adenocarcinoma lesions were observed in both groups at weeks 25 (*n* = 5) and 37 (*n* = 8), with a slight increase in TRAMP/*Phf8*‐KO. Interestingly, while NEPC lesions were identified in one TRAMP/*Phf8*‐WT mouse at week 25 (1/5) and two TRAMP mice at week 37 (2/8), none of the TRAMP/*Phf8*‐KO mice developed NEPC lesions during the same time period. Even at week 42, NEPC lesions were not found in the TRAMP/*Phf8*‐KO mice (*n* = 7, data not shown). These results indicate that knockout of *Phf8* specifically blocked NEPC development. To further confirm our H&E results, we conducted immunostaining for the adenocarcinoma marker AR and the NEPC markers SYP and CD56. AR‐positive adenocarcinomas were seen in both control and *Phf8* knockout TRAMP mice at week 25 (data not shown). However, SYP‐ and CD56‐positive NEPCs were seen only in the TRAMP control with none in *Phf8*‐KO mice at week 37 (supplementary material, Figure S2). In addition, an abdominal cavity metastasis in one mouse at week 25 and nine metastases in three mice at week 37 (three to the kidneys, one to the lung, two to the liver, two to the abdominal cavity, and one to the testis) were found in the control mice. However, no metastasis was seen in *Phf8*‐KO mice during the same time period. Consistent with results reported previously [15], almost all the metastatic lesions were NE carcinomas, as evidenced by absence of staining for AR and positive staining for SYP, CD56, and CgA (supplementary material, Figure S3A–G). Of note, the levels of PHF8 were much higher in the metastatic NE (supplementary material, Figure S3H). These results taken together suggest that PHF8 plays an important role in NEPC development but has little to do with the initiation and development of adenocarcinoma.

### PHF8 knockout impairs the NEPC signature

**Figure 2:**
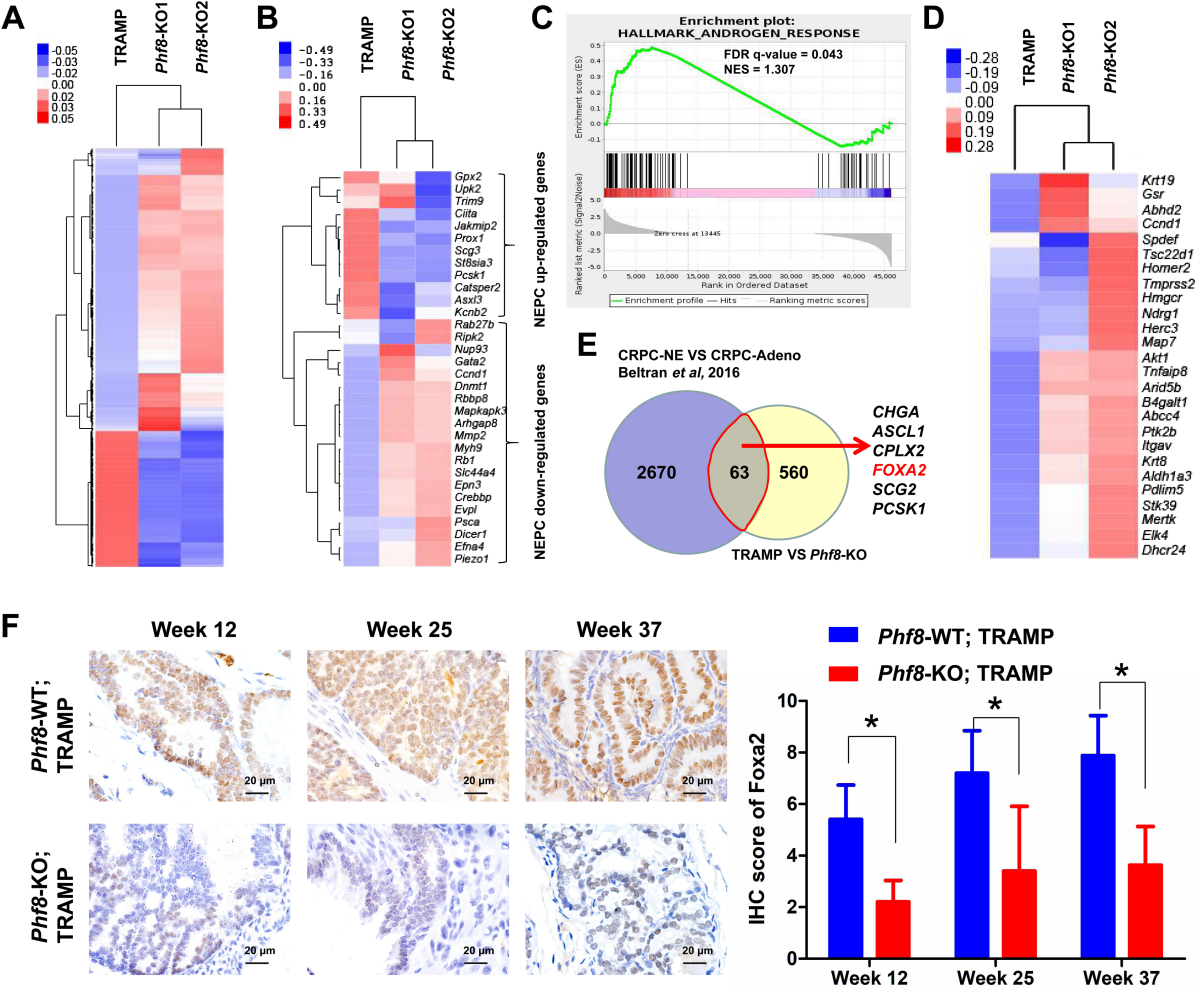
*Phf8* knockout inhibits the NEPC signature. (A) Heatmap of global altered gene expression in *Phf8*‐KO TRAMP mice compared with control TRAMP mice. (B) Heatmap of a selected gene cluster from an integrated 70‐gene NEPC classifier revealed by Beltran *et al* [11] in both groups. (C) GSEA enrichment plot of the HALLMARK_ANDROGEN_RESPONSE gene set in the above‐mentioned mice. (D) Heatmap of AR target gene expression in both groups. (E) Venn diagram revealing the commonly altered genes which were downregulated in *Phf8*‐KO mice compared with control TRAMP mice but significantly upregulated in NEPC patients, by using Beltran *et al*'s published dataset [11]. (F) Immunostaining for FOXA2 in the prostate samples from *Phf8*‐KO TRAMP mice and control TRAMP mice (non‐parametric test).

To further investigate the effect of PHF8 on NEPC development, we conducted RNA‐seq on tumor tissues derived from *Phf8*‐KO and control TRAMP mice (data deposited in GSE157621). This analysis identified 2092 differentially expressed genes (fold‐change > 2, or fold‐change < 0.5; FDR < 0.05) with 623 down‐ and 1469 up‐regulated in TRAMP/*Phf8*‐KO mice (Figure 2A). We also compared the expression of 32 genes from a panel of 70 genes considered as NEPC identifiers [11]; the results suggest that the tumors derived from *Phf8*‐KO cells more represent adenocarcinomas (Figure 2B). Results from gene set enrichment analysis (GSEA) using the AR signaling signature ‘HALLMARK_ANDROGEN_RESPONSE’ suggest that loss of PHF8 affected the AR signaling pathway (FDR *q* = 0.043, Figure 2C), as evidenced by the alteration of AR targets in *Phf8*‐knockout tumors (Figure 2D). We then conducted a bioinformatics analysis for the common altered genes which were downregulated in *Phf8*‐KO mice compared with control TRAMP mice, however significantly upregulated in NEPC patients, by using the published dataset by Beltran *et al* [11]. Of note, ASCL1 and FOXA2, two important transcriptional factors involved in NEPC development, are among them and top ranked (Figure 2E). Consistent with the RNA‐seq results, immunostaining showed that FOXA2, a transcriptional factor important for NEPC development [15, 19], was substantially reduced in tumors derived from TRAMP/*Phf8*‐KO mice (Figure 2F).

### The role of PHF8 in NEPC cell proliferation and metastasis

**Figure 3:**
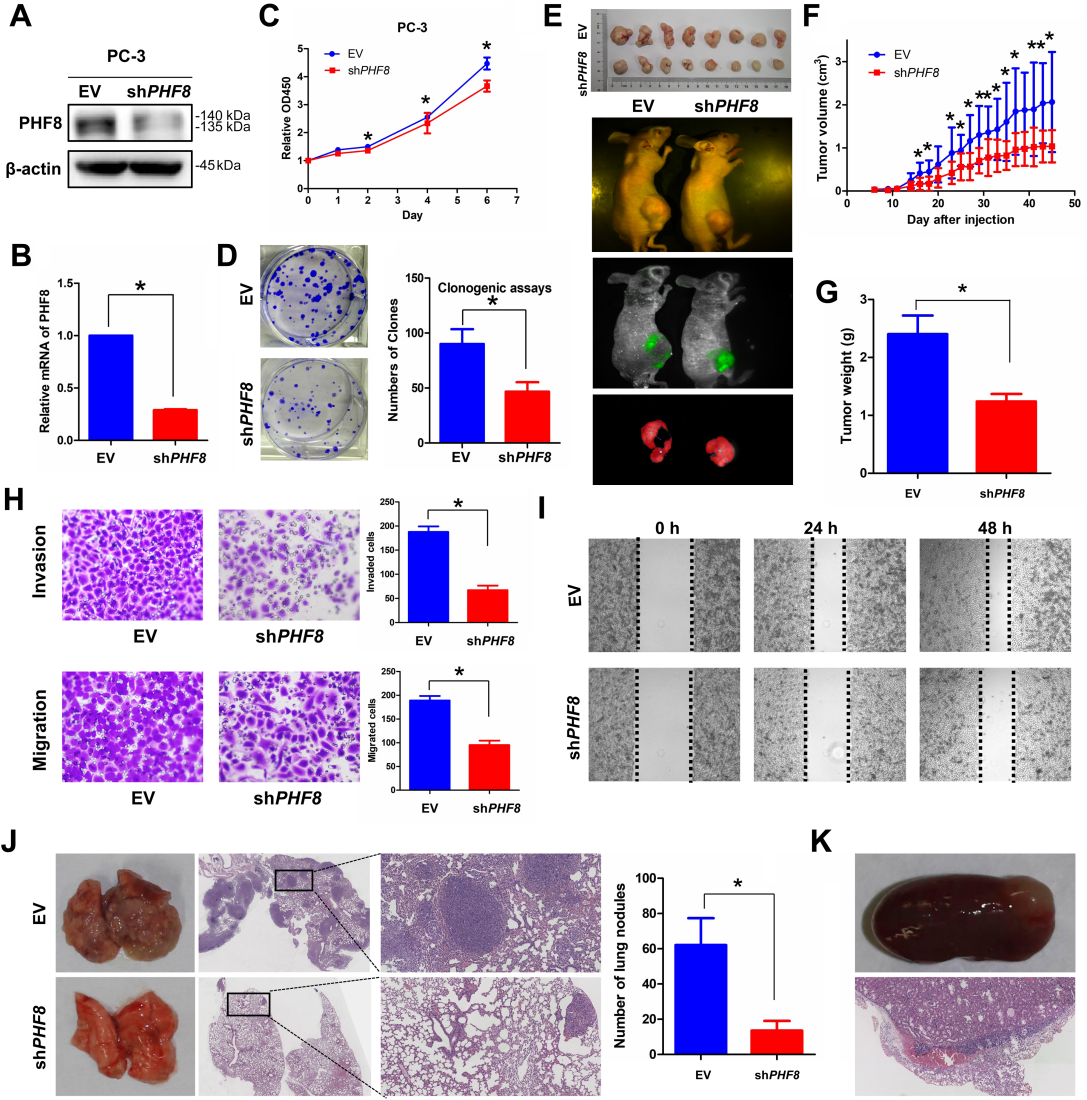
shRNA‐mediated *PHF8* knockdown inhibits the proliferation, invasion, and migration of prostate cancer. (A, B) The expression of PHF8 was examined by (A) immunoblotting and (B) RT‐qPCR after *PHF8* knockdown in PC‐3 by shRNA with empty vector as the control. (C) PC‐3 cells infected with EV or sh*PHF8* were seeded in 96‐well plates and cell numbers were estimated using CCK‐8 at days 0, 1, 2, 4, and 6. (D) PC‐3 cells with or without *PHF8* knockdown (shRNA) were seeded in six‐well plates with 1000 cells per well. Cells were fixed with methanol and stained with crystal violet. (E–G) The sizes of the tumors from mice bearing EV*‐* and sh*PHF8‐*infected PC‐3 xenografts were recorded every 2 days (E) and imaged using a whole‐body fluorescence imaging system before sacrifice (F). The tumors were collected and weighed (G). (H) PC‐3 cells with or without *PHF8* knockdown (shRNA) were seeded in 24‐well Transwell chambers with or without Matrigel and cultured for 24 h. Cell invasion and migration were estimated. (I) PC‐3 cells with or without *PHF8* knockdown (shRNA) were seeded in six‐well plates and cell migration was estimated by scratch‐wound healing assay. (J, K) PC‐3 cells with or without *PHF8* knockdown (shRNA) were injected via the tail‐vein and the lungs (J) and the kidneys (K) were harvested and sections H&E‐stained. A *t*‐test was used.

Given the fact that high‐grade neuroendocrine carcinomas including neuroblastoma, NEPC, and small‐cell carcinomas of the lung and bladder are all more aggressive [37], we wanted to determine if PHF8 plays any role in the tumor growth and invasiveness of NEPC. To do so, we knocked down PHF8 (~70%) using specific small hairpin RNAs (shRNAs) in AR‐negative, NEPC‐like PC‐3 cells (Figure 3A,B). The results in Figure 3C,D indicate that knockdown of *PHF8* inhibited both cell proliferation and colony formation. These findings were further substantiated by *in vivo* data showing that stable knockdown of *PHF8* using shRNA reduced the size (Figure 3E), volume (Figure 3F), and weight (Figure 3G) of the PC‐3 tumor xenografts in nude mice. Furthermore, the results from both Transwell and wound healing assays indicate that PHF8 is capable of enhancing migration and invasion (Figure 3H,I). Finally, we injected PC‐3 cells with (sh*PHF8*) or without (EV) *PHF8* knockdown into the tail vein and monitored the number and size of tumor nodules in the lungs. Metastasized nodules were seen in the lungs of all mice (10/10, 100%) when they were injected with PC‐3 cells infected by EV. However, not only fewer but also smaller tumor nodules were observed in the lungs of six mice (6/10, 60%) injected with PC‐3 cells infected by sh*PHF8* (Figure 3J). In addition, a kidney tumor nodule was observed in one of the controls but in none of the mice injected with PC‐3 cells infected by sh*PHF8* (Figure 3K). These data also support the notion that PHF8 plays important roles in NEPC cell proliferation, migration, and invasion. In addition, we also investigated these effects of PHF8 on the adenocarcinoma cell line (LNCaP). We found that the proliferation (supplementary material, Figure S4B), invasion (supplementary material, Figure S4C), and migration (supplementary material, Figure S4D) of LNCaP were inhibited when PHF8 was efficiently knocked down by shRNA (supplementary material, Figure S4A). Conversely, PHF8 overexpression rendered LNCaP cells resistant to anti‐androgen therapy (bicalutamide and enzalutamide treatment, supplementary material, Figure S4G,H), accompanied by increased expression of NSE (supplementary material, Figure S4E,F). These observations also suggest that the function of PHF8 is not cell type‐specific.

### PHF8 promotes NEPC development by transcriptionally upregulating FOXA2

**Figure 4:**
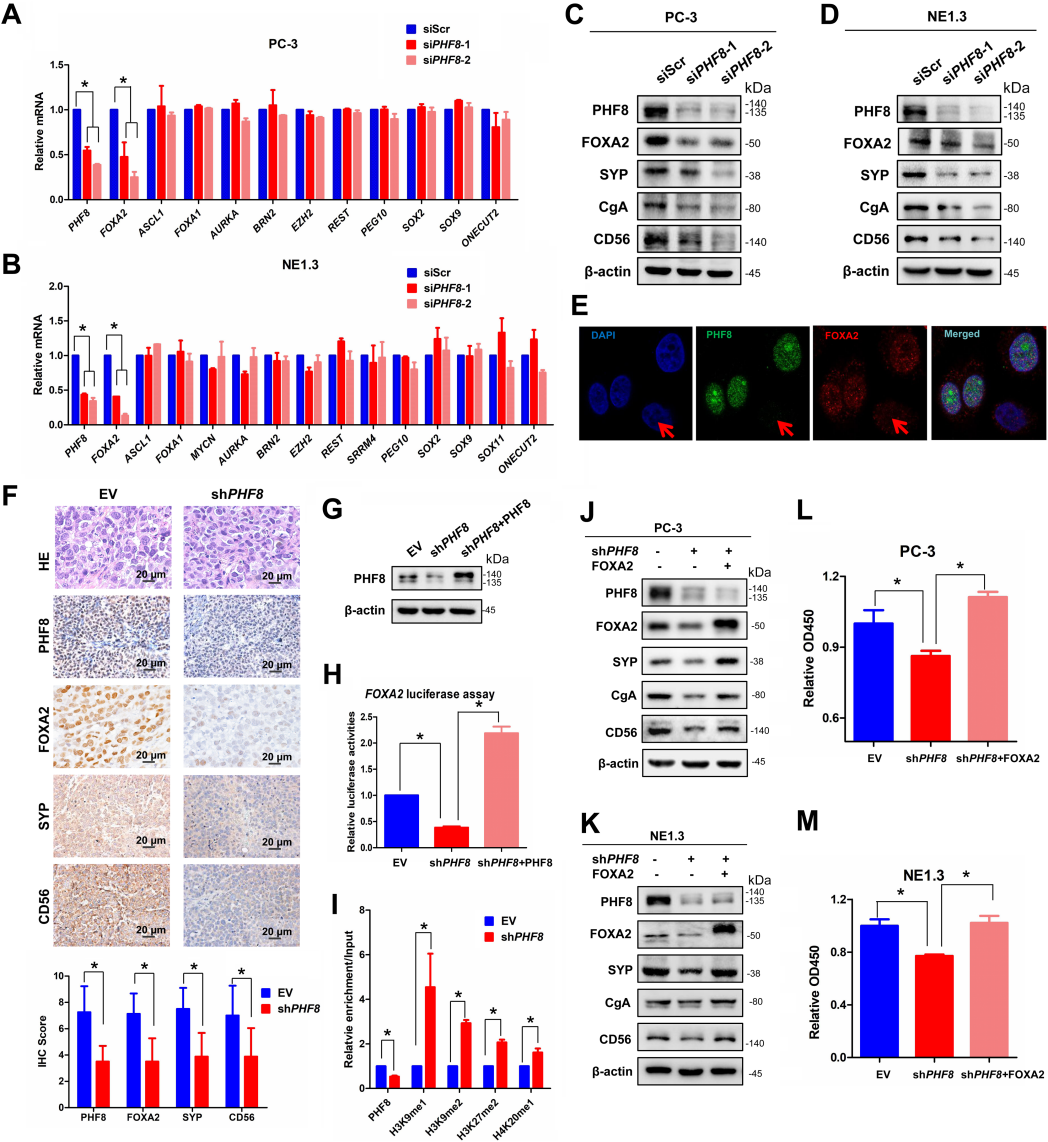
PHF8 regulates FOXA2 expression and transcription. (A, B) The mRNA levels for several transcription factors involved in NEPC development were estimated by RT‐PCR after *PHF8* knockdown by siRNA with scrambled siRNA (siScr) as the control in (A) PC‐3 and (B) NE1.3 cells (non‐parametric test). (C, D) The expression of FOXA2 and NEPC markers (SYP and CD56) was examined by immunoblotting after siRNA‐mediated *PHF8* (si*PHF8*) knockdown in (C) PC‐3 and (D) NE1.3 cells. (E) Double immunofluorescence staining analysis of PHF8 and FOXA2 was conducted after *PHF8* knockdown by using siRNA. The knockdown cell is indicated by the red arrow. (F) Immunostaining for FOXA2 and NEPC markers (SYP and CD56) in xenografts of PC‐3 cells with or without *PHF8* knockdown (shRNA) (non‐parametric test). (G, H) PC‐3 cells with or without *PHF8* knockdown (shRNA) were transfected with a *FOXA2*‐Luc construct and *Renilla* luciferase vector, as well as indicated plasmids. Cell lysates were collected to investigate the expression of PHF8 (G) and measure luciferase activity after 48 h transfection (H) (non‐parametric test). (I) PC‐3 cells with or without *PHF8* knockdown (shRNA) were subjected to CHIP assays with PHF8, H3K9me1, H3K9me2, H3K27me2, and H4K20me1 antibodies. The immunoprecipitated materials were used for qPCR analyses of the promoter regions of *FOXA2* (*t*‐test). (J, K) NEPC markers including SYP, CgA, and CD56 were estimated by western blot when FOXA2 was overexpressed in *PHF8* KD (shRNA) PC‐3 (J) and NE1.3 (K) cells. (L, M) CCK‐8 assays were used to investigate the cell viabilities after 24 h of cell culture when FOXA2 was overexpressed in *PHF8* KD (shRNA) PC‐3 (L) and NE1.3 (M) cells (non‐parametric test).

To investigate the molecular mechanism of PHF8‐regulated NEPC development, we first examined whether PHF8 regulates the expression of transcriptional factors and regulators that have been implicated in NEPC development. To do so, two prostate cancer cell lines, PC‐3 and NE1.3, with NEPC phenotype were used. When these cells were treated with *PHF8*‐specific small interference RNAs (siRNAs), the mRNA levels of both *PHF8* and *FOXA2*, but not the other genes, were significantly downregulated (Figure 4A,B). Immunoblotting analysis found that transient knockdown of *PHF8* not only markedly downregulated the levels of PHF8 and FOXA2 but also led to substantial reductions of the NEPC markers SYP, CD56, and CgA (Figure 4C,D). In addition, immunofluorescence assays showed that FOXA2 and PHF8 were co‐localized in nuclei, and reduced levels of FOXA2 were seen in *PHF8* knockdown cells (Figure 4E). Consistent with this, IHC staining also showed reduced FOXA2 and NEPC markers (SYP and CD56) in tumors derived from PC‐3 cells with shRNA‐mediated *PHF8* knockdown (Figure 4F). Since knockdown and overexpression of PHF8 down‐ and up‐regulated the *FOXA2* promoter‐regulated luciferase activity, respectively (Figure 4G,H), we conclude that PHF8 regulates FOXA2 expression transcriptionally. More importantly, the chromatin immunoprecipitation assay showed that *PHF8* knockdown also reduced PHF8 occupancy of the *FOXA2* promoter, with a concurrent increase of repressive histone markers H3K9me1, H3K9me2, H3K27me2, and H4K20me1 in the promoter region of the *FOXA2* gene (Figure 4I), suggesting that PHF8 upregulates FOXA2 expression in a demethylase activity‐dependent manner. Finally, we demonstrated that overexpressed FOXA2 in PC‐3 and NE1.3 cells infected with sh*PHF8* is capable of rescuing the NEPC markers SYP, CgA, and CD56 (Figure 4J,K), as well as the reduced cell viabilities induced by *PHF8* knockdown (Figure 4L,M), indicating that PHF8 promotes NEPC development primarily, or at least in part, by upregulating FOXA2.

### PHF8 and FOXA2 can serve as biomarkers of NEPC

**Figure 5:**
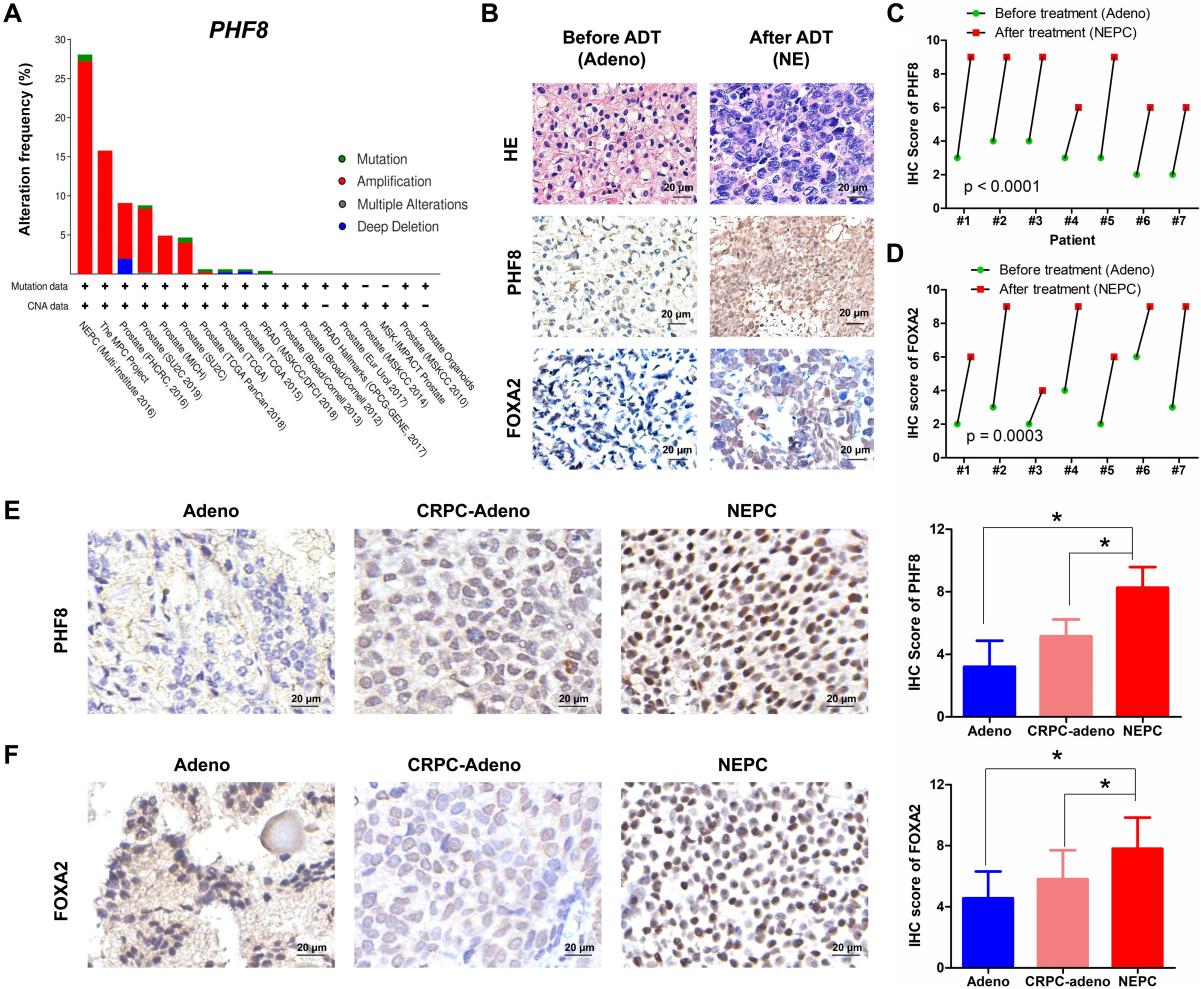
PHF8 and FOXA2 expression in adenocarcinoma and NEPC of patients' specimens. (A) Genetic changes of *PHF8* in prostate cancer from the BioPortal database. (B) Immunostaining for PHF8 and FOXA2 in the same patient before and after ADT. (C, D) Statistical results of the IHC scores for (C) PHF8 and (D) FOXA2 in B (paired *t*‐test). (E, F) Immunostaining for PHF8 (E) and FOXA2 (F) in adenocarcinoma, CRPC‐Adeno, and NEPC or NED samples (non‐parametric test).

To explore the clinical significance of PHF8 in NEPC, we first analyzed genetic alterations of *PHF8* in the publicly available large‐scale database ‘The Cancer Genome Atlas’ (TCGA; http://www.cbioportal.org) and found that ~30% patients in the NEPC cohort have *PHF8* amplification (Figure 5A), providing a molecular explanation for the elevated levels of PHF8 in NEPC. We then compared the levels of PHF8 and FOXA2 in seven paired‐prostate cancer specimens collected before and after treatments with abiraterone, enzalutamide or docetaxel. Of note, immunostaining showed that all seven tumors were adenocarcinomas before the treatment, evidenced by staining positive for both AR and PSA, and staining negative for SYP, CD56, and CgA, whereas most of them became AR‐ and PSA‐negative, and SYP‐, CD56‐, and CgA‐positive after ADT (supplementary material, Figure S5A). The representative immunostaining (Figure 5B) and the summarized results (Figure 5C,D) showed elevated levels of both PHF8 and FOXA2 in ADT‐induced NEPC. To further substantiate this notion, we compared the levels of PHF8 and FOXA2 in different patient‐derived xenograft (PDX) samples by IHC. To avoid any experimental variations, we placed NEPC‐type (characterized by staining positive for SYP, CD56, and CgA, and staining negative for AR) PDX specimens and adenocarcinoma‐type (characterized by staining negative for SYP, CD56, and CgA, and staining positive for AR; supplementary material, Figure S5B) patient samples on the same slide and conducted immunostaining with antibodies against PHF8 and FOXA2. Supplementary material, Figure S5 shows that the levels of both PHF8 and FOXA2 in NEPC lesions are much higher than those in the adenocarcinoma. Similarly, the levels of PHF8 and FOXA2 in NEPC were also higher than those in CRPC when similar experiments were conducted with the NEPC PDX (LTL‐545) and the CRPC PDX tissues (LTL‐313HR, characterized by staining negative for SYP, CD56, and CgA, and staining positive for AR). Finally, the IHC scores of the PHF8 and FOXA2 staining in 59 adenocarcinoma, 13 CRPC‐Adeno, and 10 NEPC or NED specimens indicate that expression of PHF8 (Figure 5E) and FOXA2 (Figure 5F) in NEPC tissues is much higher than in adenocarcinoma (Adeno) and CRPC‐Adeno tissues. These findings are in concordance with the expression of PHF8 and FOXA2 found in NEPC and CRPC samples in the public dataset of Beltran *et al* [11] (supplementary material, Figure S6A,B). Furthermore, the expression of PHF8 positively correlated with that of FOXA2 (supplementary material, Figure S6C). In addition, we investigated the association of the expression of PHF8 and FOXA2 with pathological grade (Gleason scores) in 42 specimens with prostate adenocarcinoma (supplementary material, Figure S6D–H). Our results revealed that compared with lower‐grade tumors (Gleason score less than or equal to 7), the levels of both PHF8 (supplementary material, Figure S6D–F) and FOXA2 (supplementary material, Figure S6E–G) were significantly higher in tumors with higher Gleason scores (>7). More importantly, a positive correlation between the levels of PHF8 and FOXA2 in prostate adenocarcinoma samples is revealed by correlation analyses (supplementary material, Figure S6H) and this finding is further substantiated by the heatmap (supplementary material, Figure S6I), which reflects the relationship between the levels of PHF8 and FOXA2 as well as their expression and the degree of malignancies. Since the levels of PHF8 and FOXA2 are higher in NEPC and positively correlated with each other, we propose that the elevated levels of PHF8 and FOXA2 can either individually or in combination serve as NEPC diagnostic biomarkers.

## Discussion

**Figure 6:**
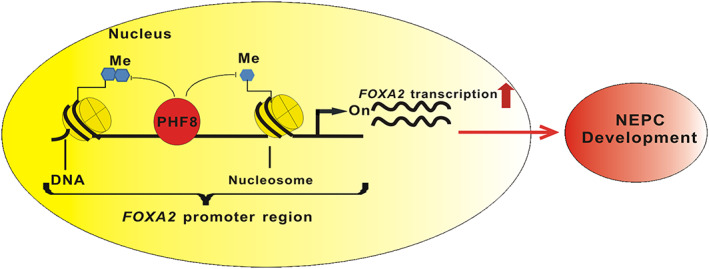
Schematic illustration of the proposed model in which PHF8 regulates FOXA2 and NEPC development.

Our current research identified a previously unknown PHF8/FOXA2 axis in prostate cancer as well as its role in NEPC development. Since knocking out *Phf8* had minimal effect on the initiation and progression of prostate adenocarcinoma but abrogated both the initiation and the metastasis of NEPC, we conclude that PHF8 plays a unique role in NEPC development. Based on the fact that PHF8 upregulates FOXA2 transcriptionally by demethylating and removing the repressive histone marks in the promoter region of the *FOXA2* gene and that overexpressed FOXA2 is capable of rescuing NEPC phenotype, we proposed a working model to illustrate the role of the PHF8/FOXA2 axis in NEPC development (Figure 6). Since both PHF8 and FOXA2 are highly expressed in NEPC cells, we suggest that the levels of PHF8 and FOXA2 can either individually or in combination serve as NEPC biomarkers, and that targeting either PHF8 or FOXA2 could be potential therapeutic strategies for NEPC treatment. However, the prognostic values of PHF8 and FOXA2 in NEPC patients need to be further investigated by using larger cohorts of samples.

Multiple factors including AURKA, MYCN, BRN2, SRRM4, REST, EZH2, and FOXA2 have been implicated in the development of NEPC. For example, both AURKA and MYCN are elevated in NEPC and they function cooperatively to promote NEPC [13]. It is also well established that AR‐repressed BRN2 can induce neuroendocrine differentiation in both castration‐ and enzalutamide‐resistant prostate cancer models [14]. The RNA splicing factor SRRM4 is capable of driving adenocarcinoma cell progression toward NEPC with concurrent upregulation of NEPC‐specific biomarkers [38]. On the other hand, both REST and EZH2 are involved in NEPC progression by epigenetic modification of the genome [17, 18]. Although FOXA2 appeared to be involved in NEPC development, the underlying molecular mechanism was unknown. By using different model systems including combined *Phf8* knockout and the TRAMP mouse model, NEPC cell lines, NEPC PDX and patients' cancer tissues, we established the PHF8/FOXA2 axis and illustrated its role in NEPC development. We noticed that results from previous research using LNCaP cells suggest that PHF8 promotes CRPC but suppresses neuroendocrine differentiation [36]. The discrepancy between our findings and previously reported results could be the use of different cell lines and experimental systems. In the TRAMP model system, PHF8 plays an indispensable role in NEPC development, although knocking out *Phf8* has minimal effect on the development of adenocarcinoma. Therefore, the mechanisms in androgen depletion‐induced NED of LNCaP cells could be different from those in NEPC development in the TRAMP model and further research will reconcile these differences. The TRAMP mouse model has some inherent limitations because the viral antigens are not naturally associated with human prostate cancer and the model would develop some extensive neuroendocrine tumors spontaneously different from the development of human NEPC [5]. Nevertheless, the TRAMP model is still a useful means to investigate the molecular mechanism underlying the progression of NEPC because the formation of NEPC in TRAMP mice partially resulted from the inactivation of p53 and RB1, crucial events happening during the NED of human prostate cancer [12].

Although we demonstrated a critical role of PHF8 in NEPC development, there are many unanswered questions. For example, increased expression of PHF8 enhanced NEPC development by epigenetically upregulating the transcription factor FOXA2, but the cause of PHF8 upregulation is elusive. It has been reported recently that lineage tracing experiments in TRAMP mice showed that NEPC might originate from the p63‐positive basal cells instead of the pre‐existing adenocarcinoma cells [7]. Whether upregulated PHF8 has anything to do with these basal cells is unknown. On the other hand, *Phf8* knockout completely inhibited the NEPC development in TRAMP mice without affecting the development of CK5‐positive basal cells in C57 mice; whether PHF8 affects malignant transformation of basal cells needs to be further investigated. Furthermore, PHF8 upregulates FOXA2 by erasing repressive histone markers. It is unclear if PHF8 interacts with the chromosome directly or through other transcriptional factors indirectly. Further analyses using tools from the GeneCards website (https://www.genecards.org/) suggest that the transcriptional factors TCF4 and REST could be involved in the regulation of FOXA2. It has also been reported that β‐catenin is recruited to the TCF/LEF‐binding site in the promoter region of *FOXA2* [39] and our preliminary data indicate that PHF8 can complex with β‐catenin (data not shown). In addition, it has been reported that PHF8 and REST can co‐occupy the same chromosome regions. These lines of evidence suggest that PHF8 could upregulate FOXA2 by complexing with either REST or β‐catenin/TCF4 and subsequently erasing the repressive histone marks on the promoter region of the *FOXA2* gene.

In summary, by using transgenic and knockout mice, *in vitro* cell lines, and *in vivo* xenografts, as well as NEPC PDX and patients' tissue samples, we revealed a critical role of the epigenetic regulator PHF8 in NEPC development, rendering PHF8 a potential therapeutic target for the deadly NEPC.

## Author contributions statement

JJ and JQ had full access to all the data in the study and take responsibility for the integrity of the data and the accuracy of the data analysis. JJ and JQ were responsible for study concept and design. QL, JP, LAW, ZH, JX, XY, QX, YH, TT, GL and DT acquired data. QL, JP, LAW, WL and LFW analyzed and interpreted data. QL drafted the manuscript. JJ, JQ, DZ, WL and LFW critically revised the manuscript for important intellectual content. QL, GL and DT carried out statistical analysis. JJ and QL obtained funding. JJ and JQ supervised the study.

## Supporting information


**Supplementary figure legends**
**Figure S1.** The effect of *Phf8* knockout on the development of prostate in C57 mice
**Figure S2.** Immunostaining for an adenocarcinoma marker (AR) and NEPC markers (SYP and CD56), as well as PHF8 and Large‐T, in *Phf8*‐WT and *Phf8*‐KO TRAMP mice at week 37
**Figure S3.** Immunostaining for an adenocarcinoma marker (AR) and NEPC markers (SYP and CD56), as well as PHF8 and Large‐T, in metastatic lesions of TRAMP mice
**Figure S4.** The effects of *PHF8* knockdown or overexpression on the proliferation, invasion, and migration, as well as response to anti‐androgen therapy, of LNCaP cells
**Figure S5.** Immunostaining of patient samples and cell lines
**Figure S6.** Comparison of expression (*t*‐test) and correlation (Pearson's test) of PHF8 and FOXA2 in the published dataset of Beltran *et al* [11]

**Table S1.** Clinical information of the patients with NEPC or NED
**Table S2.** Clinical information of the patients with CRPC‐Adeno
**Table S3.** Sequences of the primers used

## Data Availability

The raw data from our RNA‐seq study can be downloaded from GSE157621 (https://www.ncbi.nlm.nih.gov/geo/query/acc.cgi?acc=GSE157621).
