# Supplementary material for: Histone demethylase PHF8 drives neuroendocrine prostate cancer progression by epigenetically upregulating FOXA2
Source: J Pathol. 2020 Nov 5;253(1):106–18. doi: 10.1002/path.5557 (PMC7756255; doi:10.1002/path.5557)
Supplement: Supplementary file 1 — Supplementary figure legends Figure S1. The effect of Phf8 knockout on the development of prostate in C57 mice Figure S2. Immunostaining for an adenocarcinoma marker (AR) and NEPC markers (SYP and CD56), as well as PHF8 and Large‐T, in Phf8‐WT and Phf8‐KO TRAMP mice at week 37 Figure S3. Immunostaining for an adenocarcinoma marker (AR) and NEPC markers (SYP and CD56), as well as PHF8 and Large‐T, in metastatic lesions of TRAMP mice Figure S4. The effects of PHF8 knockdown or overexpression on the proliferation, invasion, and migration, as well as response to anti‐androgen therapy, of LNCaP cells Figure S5. Immunostaining of patient samples and cell lines Figure S6. Comparison of expression (t‐test) and correlation (Pearson's test) of PHF8 and FOXA2 in the published dataset of Beltran et al [11] [file PATH-253-106-s001.zip › path5557-sup-SuppFigLegs.docx]

**Histone demethylase PHF8 drives neuroendocrine prostate cancer progression by epigenetically upregulating FOXA2**

Q Liu, J Pang, L-A Wang, *et al. J Pathol* DOI: 10.1002/path.5557

**Supplementary figure legends**

**Figure S1.** The effect of *Phf8* knockout on the development of prostate in C57 mice. (A–C) H&E-stained sections of representative anterior prostate, dorsal-lateral prostate, and ventral prostate in *Phf8*-WT C57 mouse and *Phf8*-KO C57 mouse at the indicated time points. (D–F) Double immunofluorescence staining analysis for CK5 and CK18 was conducted in the above-mentioned mice.

**Figure S2.** Immunostaining for an adenocarcinoma marker (AR) and NEPC markers (SYP and CD56), as well as PHF8 and Large-T, in *Phf8*-WT and *Phf8*-KO TRAMP mice at week 37.

**Figure S3.** Immunostaining for an adenocarcinoma marker (AR) and NEPC markers (SYP and CD56), as well as PHF8 and Large-T, in metastatic lesions of TRAMP mice.

**Figure S4.** The effects of *PHF8* knockdown or overexpression on the proliferation, invasion, and migration, as well as response to anti-androgen therapy, of LNCaP cells. (A) The expression of PHF8 was examined by immunoblotting after *PHF8* knockdown in LNCaP cells by shRNA with empty vector as the control. (B) LNCaP cells infected with EV or sh*PHF8* were seeded in 96-well plates and cell numbers were estimated using CCK-8 at days 0, 1, 2, 3, and 4. (C, D) LNCaP cells with or without *PHF8* knockdown (shRNA) were seeded in 24-well Transwell chambers with (C) or without (D) Matrigel and cultured for 48 h. Cell invasion and migration were estimated (*t*-test). (E, F) The expression of PHF8 and NSE was examined by (E) RT-qPCR and (F) immunoblotting after PHF8 overexpression in LNCaP cells with empty vector as the control (*t*-test). (G, H) LNCaP cells with or without PHF8 overexpression were seeded in six-well plates with 2000 cells per well. Cells were fixed with methanol and stained with crystal violet. The colonies were counted (*t*-test).

**Figure S5.** Immunostaining of patient samples and cell lines. (A) Immunostaining for AR, PSA, SYP, CD56, and CgA in the same patient before and after ADT. (B) Immunostaining for AR, SYP, CD56, CgA, FOXA2, and PHF8 in a slide including one patient with prostate adenocarcinoma and a neuroendocrine PDX tissue line, LTL-545. (C) Immunostaining for AR, SYP, CD56, CgA, FOXA2, and PHF8 in a slide including a CRPC PDX tissue line, LTL-331HR, and a neuroendocrine PDX tissue line, LTL-545.

**Figure S6.** Comparison of expression (*t*-test) and correlation (Pearson’s test) of PHF8 and FOXA2 in the published dataset ‘Beltran *et al* 2016’ [11] (A–C) and our tissue microarray of prostate adenocarcinoma. (D, E) Representative immunostaining of PHF8 (D) and FOXA2 (E) conducted on 42 human prostate adenocarcinoma TMA specimens. (F, G) IHC scores for PHF8 (F) and FOXA2 (G) were compared among different Gleason grades (non-parametric test). (H) Correlation analyses (Pearson’s test) of the levels of PHF8 and FOXA2. (I) Log-transformed data of the IHC score were used to make a heatmap.
