# Supplementary figures and images for: Histone demethylase PHF8 drives neuroendocrine prostate cancer progression by epigenetically upregulating FOXA2
Source: J Pathol. 2020 Nov 5;253(1):106–18. doi: 10.1002/path.5557 (PMC7756255; doi:10.1002/path.5557)

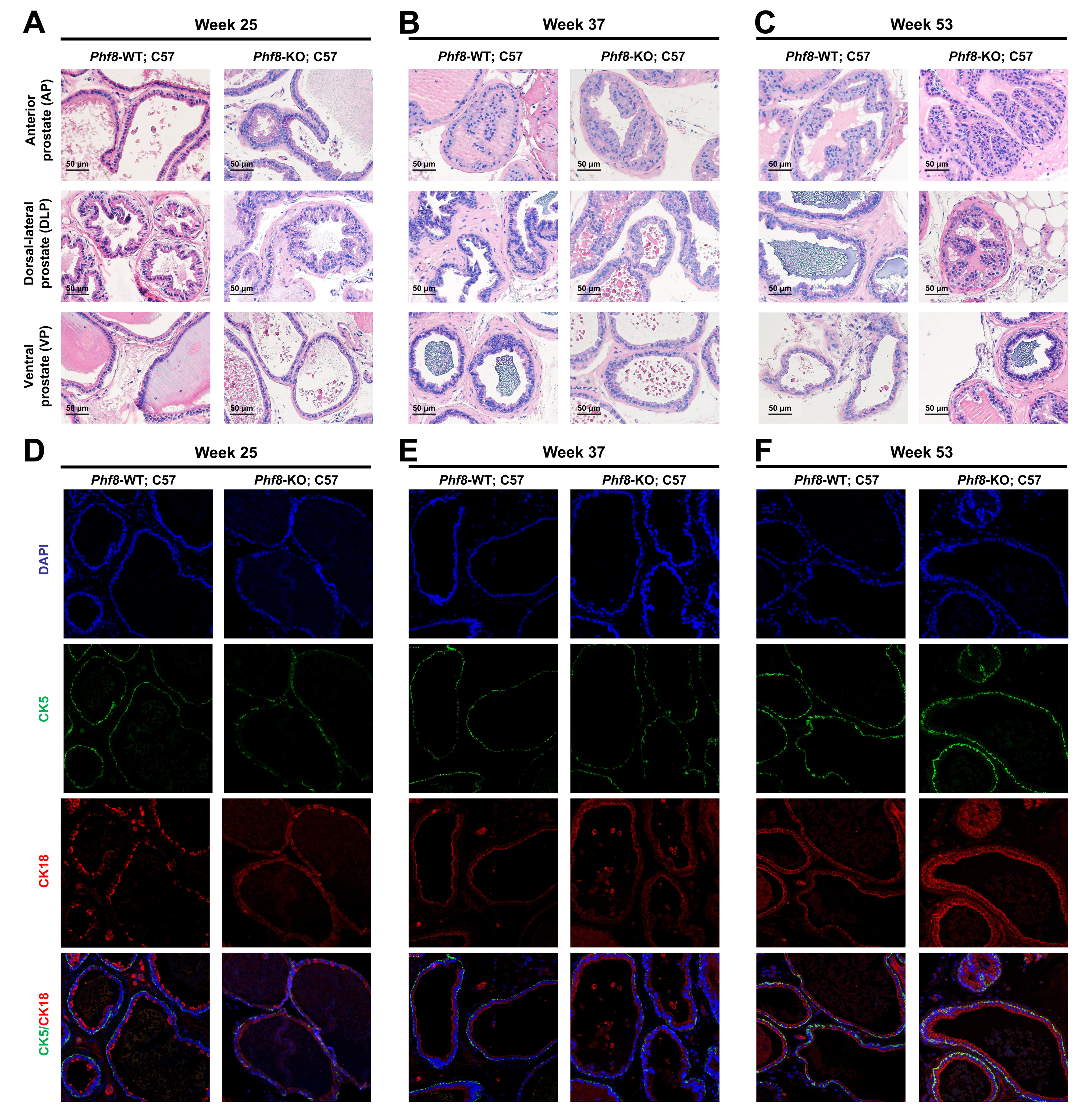

Supplement: Supplementary file 1 — Supplementary figure legends Figure S1. The effect of Phf8 knockout on the development of prostate in C57 mice Figure S2. Immunostaining for an adenocarcinoma marker (AR) and NEPC markers (SYP and CD56), as well as PHF8 and Large‐T, in Phf8‐WT and Phf8‐KO TRAMP mice at week 37 Figure S3. Immunostaining for an adenocarcinoma marker (AR) and NEPC markers (SYP and CD56), as well as PHF8 and Large‐T, in metastatic lesions of TRAMP mice Figure S4. The effects of PHF8 knockdown or overexpression on the proliferation, invasion, and migration, as well as response to anti‐androgen therapy, of LNCaP cells Figure S5. Immunostaining of patient samples and cell lines Figure S6. Comparison of expression (t‐test) and correlation (Pearson's test) of PHF8 and FOXA2 in the published dataset of Beltran et al [11] [file PATH-253-106-s001.zip › path5557-sup-FigureS1.tif]

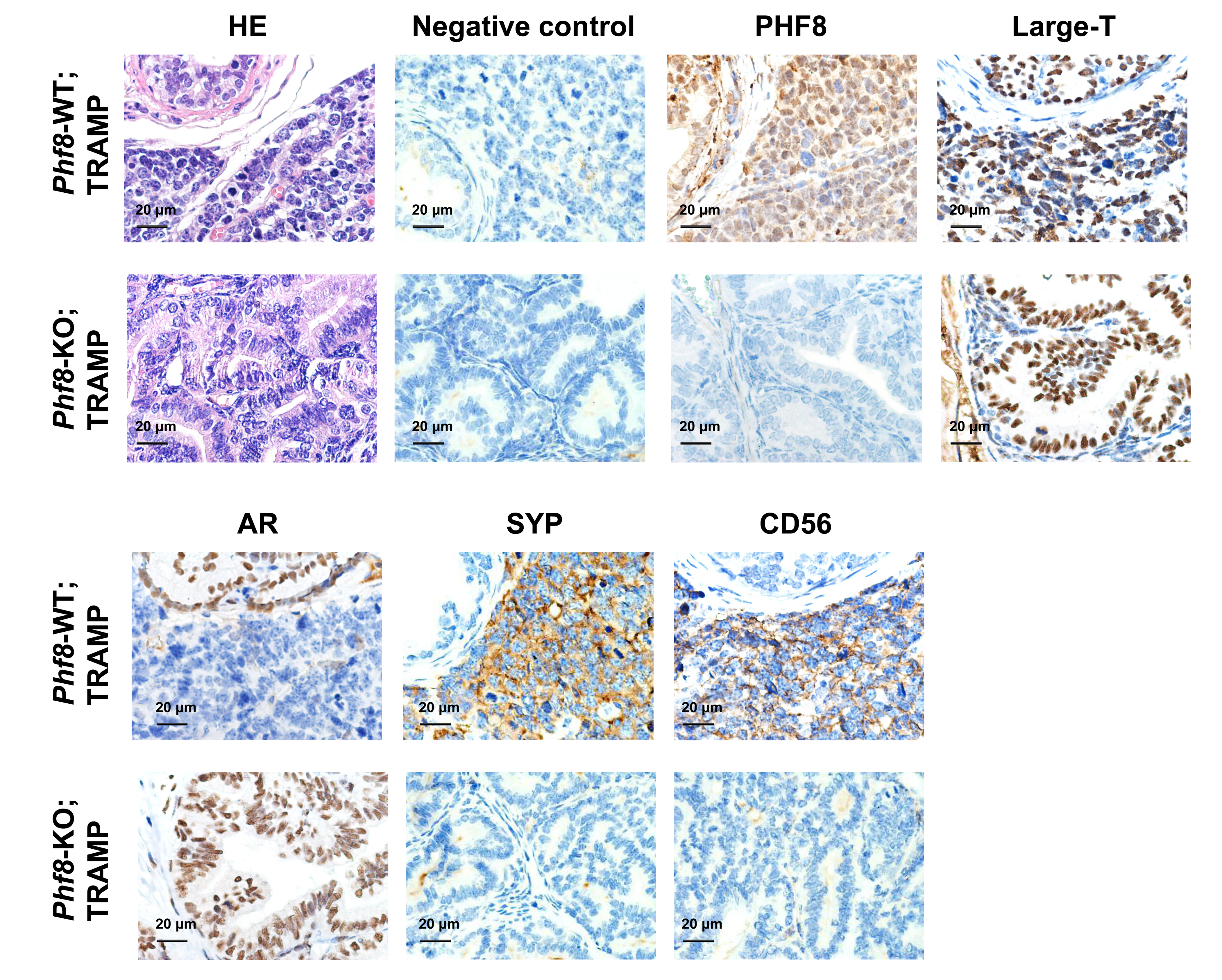

Supplement: Supplementary file 1 — Supplementary figure legends Figure S1. The effect of Phf8 knockout on the development of prostate in C57 mice Figure S2. Immunostaining for an adenocarcinoma marker (AR) and NEPC markers (SYP and CD56), as well as PHF8 and Large‐T, in Phf8‐WT and Phf8‐KO TRAMP mice at week 37 Figure S3. Immunostaining for an adenocarcinoma marker (AR) and NEPC markers (SYP and CD56), as well as PHF8 and Large‐T, in metastatic lesions of TRAMP mice Figure S4. The effects of PHF8 knockdown or overexpression on the proliferation, invasion, and migration, as well as response to anti‐androgen therapy, of LNCaP cells Figure S5. Immunostaining of patient samples and cell lines Figure S6. Comparison of expression (t‐test) and correlation (Pearson's test) of PHF8 and FOXA2 in the published dataset of Beltran et al [11] [file PATH-253-106-s001.zip › path5557-sup-FigureS2.tif]

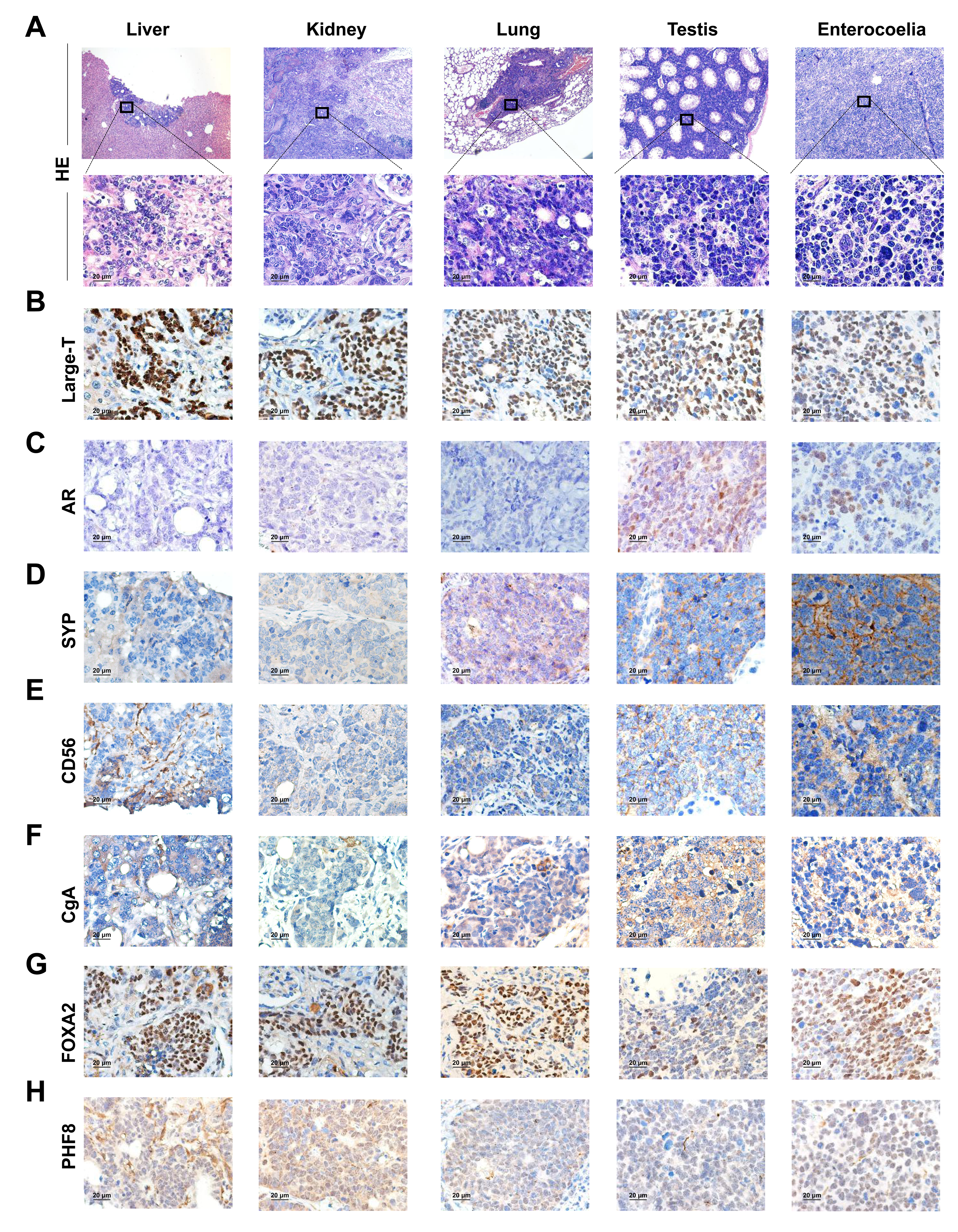

Supplement: Supplementary file 1 — Supplementary figure legends Figure S1. The effect of Phf8 knockout on the development of prostate in C57 mice Figure S2. Immunostaining for an adenocarcinoma marker (AR) and NEPC markers (SYP and CD56), as well as PHF8 and Large‐T, in Phf8‐WT and Phf8‐KO TRAMP mice at week 37 Figure S3. Immunostaining for an adenocarcinoma marker (AR) and NEPC markers (SYP and CD56), as well as PHF8 and Large‐T, in metastatic lesions of TRAMP mice Figure S4. The effects of PHF8 knockdown or overexpression on the proliferation, invasion, and migration, as well as response to anti‐androgen therapy, of LNCaP cells Figure S5. Immunostaining of patient samples and cell lines Figure S6. Comparison of expression (t‐test) and correlation (Pearson's test) of PHF8 and FOXA2 in the published dataset of Beltran et al [11] [file PATH-253-106-s001.zip › path5557-sup-FigureS3.tif]

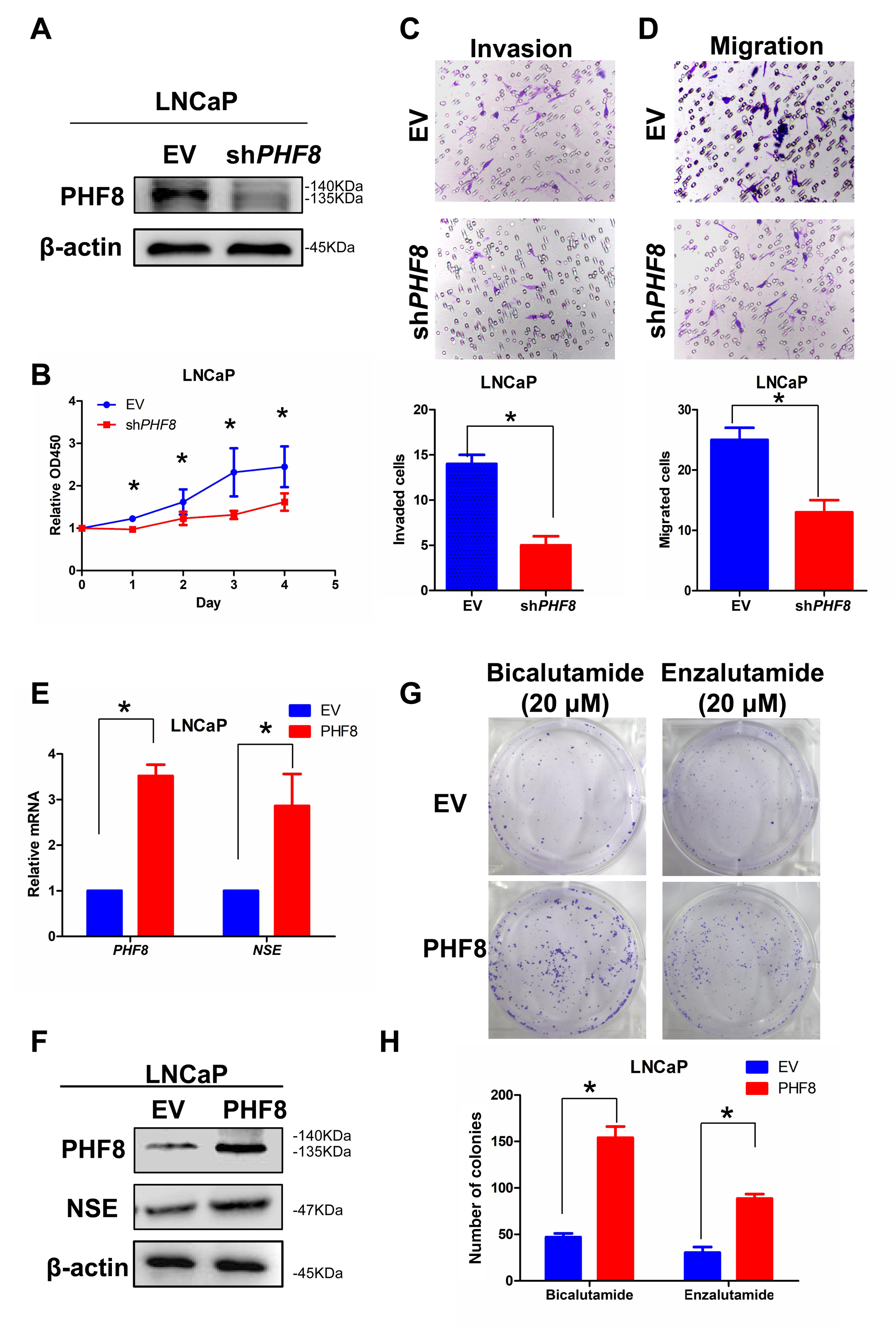

Supplement: Supplementary file 1 — Supplementary figure legends Figure S1. The effect of Phf8 knockout on the development of prostate in C57 mice Figure S2. Immunostaining for an adenocarcinoma marker (AR) and NEPC markers (SYP and CD56), as well as PHF8 and Large‐T, in Phf8‐WT and Phf8‐KO TRAMP mice at week 37 Figure S3. Immunostaining for an adenocarcinoma marker (AR) and NEPC markers (SYP and CD56), as well as PHF8 and Large‐T, in metastatic lesions of TRAMP mice Figure S4. The effects of PHF8 knockdown or overexpression on the proliferation, invasion, and migration, as well as response to anti‐androgen therapy, of LNCaP cells Figure S5. Immunostaining of patient samples and cell lines Figure S6. Comparison of expression (t‐test) and correlation (Pearson's test) of PHF8 and FOXA2 in the published dataset of Beltran et al [11] [file PATH-253-106-s001.zip › path5557-sup-FigureS4.tif]

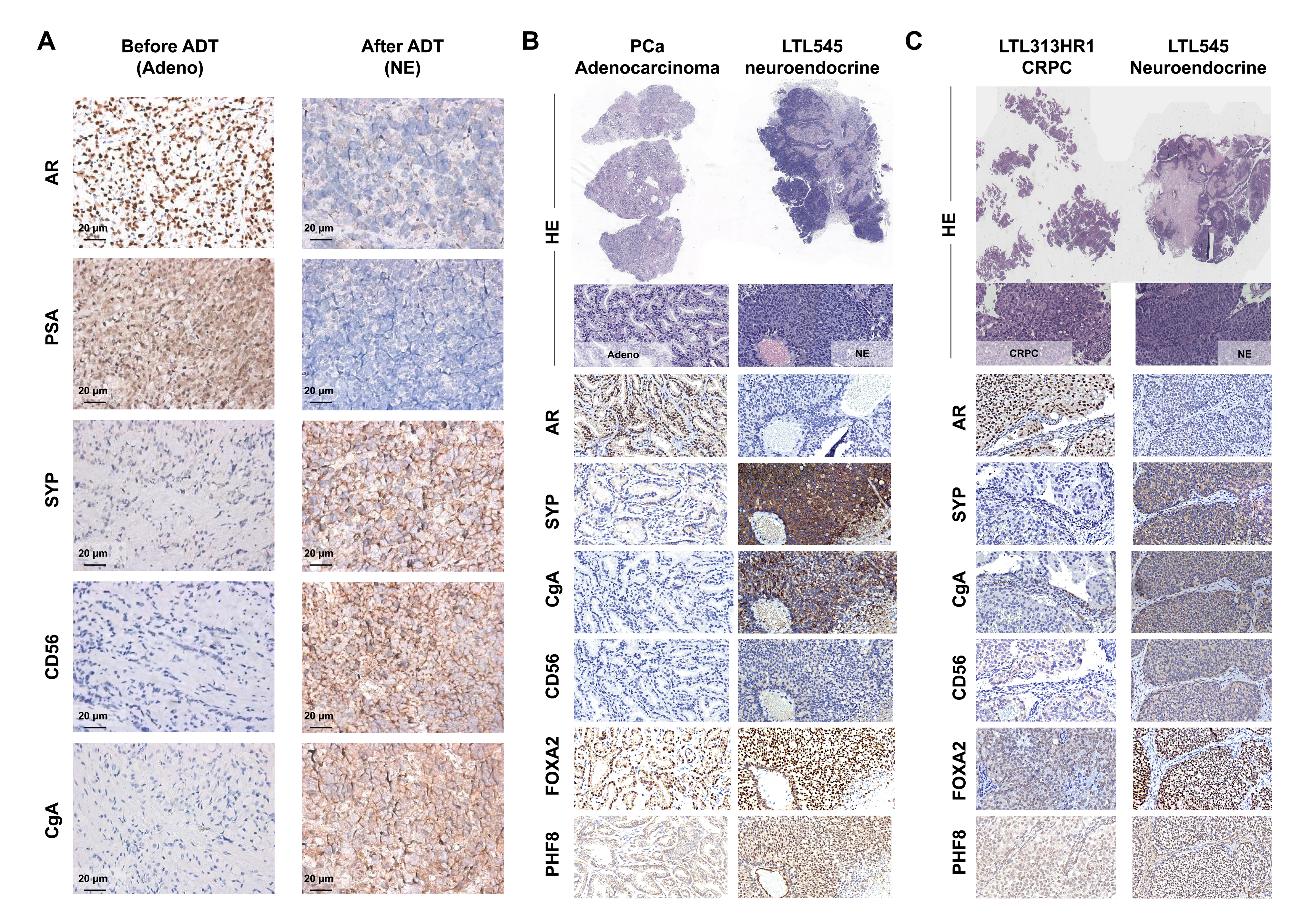

Supplement: Supplementary file 1 — Supplementary figure legends Figure S1. The effect of Phf8 knockout on the development of prostate in C57 mice Figure S2. Immunostaining for an adenocarcinoma marker (AR) and NEPC markers (SYP and CD56), as well as PHF8 and Large‐T, in Phf8‐WT and Phf8‐KO TRAMP mice at week 37 Figure S3. Immunostaining for an adenocarcinoma marker (AR) and NEPC markers (SYP and CD56), as well as PHF8 and Large‐T, in metastatic lesions of TRAMP mice Figure S4. The effects of PHF8 knockdown or overexpression on the proliferation, invasion, and migration, as well as response to anti‐androgen therapy, of LNCaP cells Figure S5. Immunostaining of patient samples and cell lines Figure S6. Comparison of expression (t‐test) and correlation (Pearson's test) of PHF8 and FOXA2 in the published dataset of Beltran et al [11] [file PATH-253-106-s001.zip › path5557-sup-FigureS5.tif]

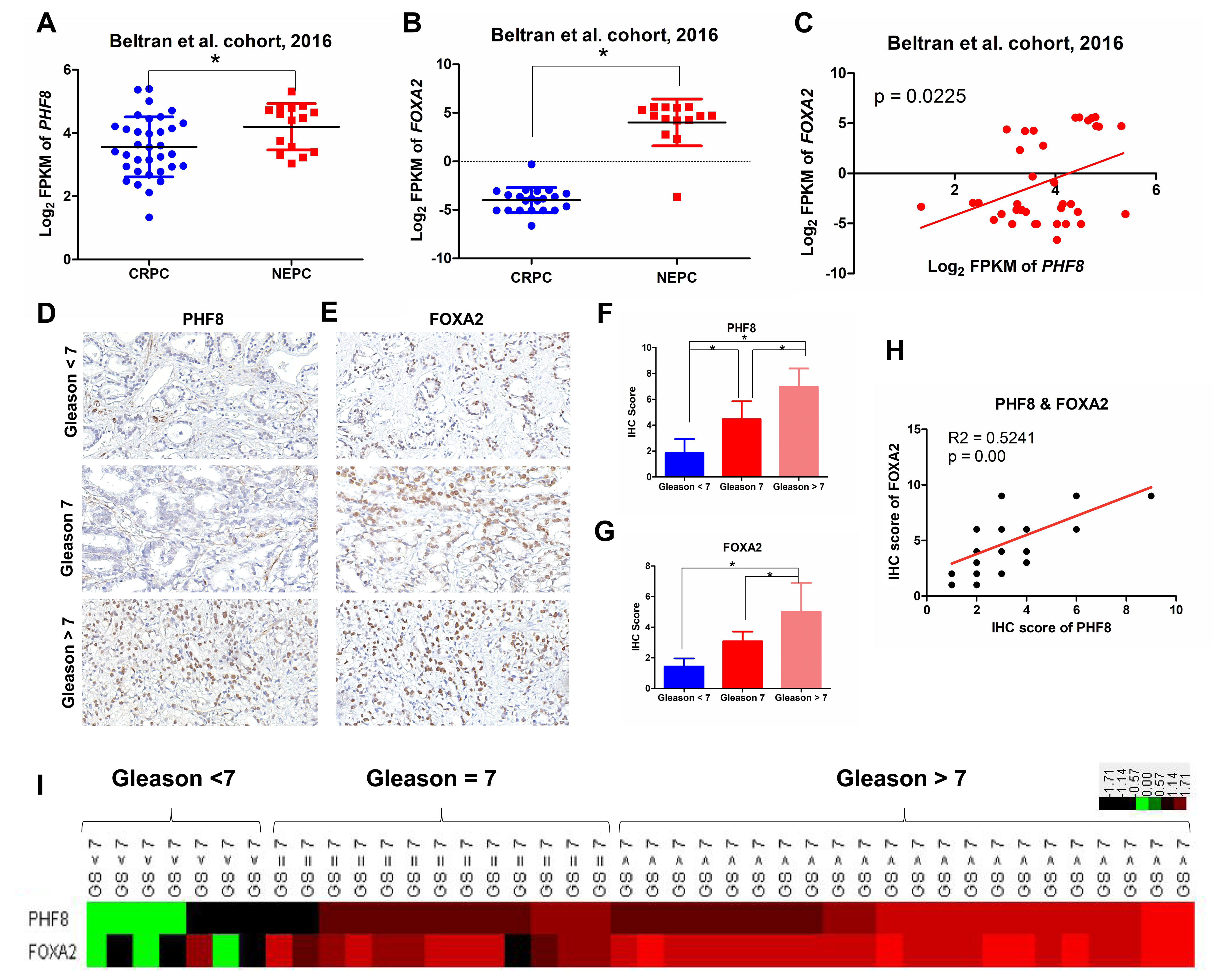

Supplement: Supplementary file 1 — Supplementary figure legends Figure S1. The effect of Phf8 knockout on the development of prostate in C57 mice Figure S2. Immunostaining for an adenocarcinoma marker (AR) and NEPC markers (SYP and CD56), as well as PHF8 and Large‐T, in Phf8‐WT and Phf8‐KO TRAMP mice at week 37 Figure S3. Immunostaining for an adenocarcinoma marker (AR) and NEPC markers (SYP and CD56), as well as PHF8 and Large‐T, in metastatic lesions of TRAMP mice Figure S4. The effects of PHF8 knockdown or overexpression on the proliferation, invasion, and migration, as well as response to anti‐androgen therapy, of LNCaP cells Figure S5. Immunostaining of patient samples and cell lines Figure S6. Comparison of expression (t‐test) and correlation (Pearson's test) of PHF8 and FOXA2 in the published dataset of Beltran et al [11] [file PATH-253-106-s001.zip › path5557-sup-FigureS6.tif]
