# Supplementary material for: Histone demethylase PHF8 drives neuroendocrine prostate cancer progression by epigenetically upregulating FOXA2
Source: J Pathol. 2020 Nov 5;253(1):106–18. doi: 10.1002/path.5557 (PMC7756255; doi:10.1002/path.5557)
Supplement: Supplementary file 2 — Table S1. Clinical information of the patients with NEPC or NED Table S2. Clinical information of the patients with CRPC‐Adeno Table S3. Sequences of the primers used [file PATH-253-106-s002.docx]

**Histone demethylase PHF8 drives neuroendocrine prostate cancer progression by epigenetically upregulating FOXA2**

Q Liu, J Pang, L-A Wang, *et al. J Pathol* DOI: 10.1002/path.5557

**Supplementary Tables S1–S3**

**Table S1.** Clinical information of the patients with NEPC or NED

| **Patient number** | **Date when collecting specimens** | **Age** | **PSA** | **Gleason score** | **Pathological results** | **Strategy of androgen deprivation therapy** |
| --- | --- | --- | --- | --- | --- | --- |
| 1 | 12/2/2013 | 64 | >100 | 5 + 4 | Adenocarcinoma | GnRHa + Bicalutamide |
|  | 10/27/2014 | 65 | 7.25 |  | NEPC |  |
| 2 | 9/22/2015 | 65 | >100 | 4 + 5 | Adenocarcinoma | GnRHa + Bicalutamide |
|  | 11/5/2015 | 65 | 0.24 |  | NED |  |
| 3 | 9/5/2007 | 66 | 11.68 | 4 + 5 | Adenocarcinoma | Surgery for castration |
|  | 2/8/2012 | 71 | 0.58 |  | NEPC |  |
| 4 | 3/19/2019 | 69 | 8.69 | 5 + 5 | Adenocarcinoma | GnRHa + Bicalutamide |
|  | 4/30/2019 | 69 | 0.13 |  | NED |  |
| 5 | 8/31/2010 | 65 | 24.02 | 3 + 4 | Adenocarcinoma | GnRHa + Bicalutamide |
|  | 10/20/2019 | 74 | 0.98 |  | NEPC |  |
| 6 | 6/24/2016 | 68 | >154 | 5 + 4 | Adenocarcinoma | GnRHa + Enzalutamide |
|  | 11/5/2019 | 71 | 0.22 |  | NEPC |  |
| 7 | 9/18/2018 | 62 | >158 | 5 + 5 | Adenocarcinoma | GnRHa + Bicalutamide  + Docetaxel |
|  | 12/11/2019 | 64 | 7.58 |  | NED |  |

**Table S2.** Clinical information of the patients with CRPC-Adeno

| **Patient number** | **Age at diagnosis** | **PSA at diagnosis (μg/l)** | **Gleason score** | **Clinical TNM stage** | **Strategy of androgen deprivation therapy** | **Time from diagnosis to progression to CRPC (months)** |
| --- | --- | --- | --- | --- | --- | --- |
| 1 | 60 | 38.41 | 5 + 4 | T4N1M1b | GnRHa + Bicalutamide | 17 |
| 2 | 74 | 149 | 4 + 4 | T3bNxM1b | GnRHa + Bicalutamide | 6 |
| 3 | 61 | 100 | 3 + 4 | T3aNxM1b | GnRHa + Bicalutamide | 12 |
| 4 | 82 | 14 | 5 + 5 | T4N0M0 | GnRHa + Bicalutamide | 9 |
| 5 | 76 | 149 | 4 + 4 | T3aNxM1b | GnRHa + Bicalutamide | 19 |
| 6 | 53 | 103 | 5 + 5 | T4N1M0 | GnRHa + Bicalutamide | 6 |

**Table S3.** Sequences of the primers used

| **RT-qPCR primers** | |
| --- | --- |
| *PHF8* | TTCATGGCAGTTGTGTTGGTG |
|  | TGCAAGACTTCACAGTTGGG |
| *NSE* | AACTGGAGGGTGCATTATCTTTC |
|  | CAGTACAACAATGGGCTGTAGG |
| *FOXA2* | GGAGCAGCTACTATGCAGAGC |
|  | CGTGTTCATGCCGTTCATCC |
| *GAPDH* | AAGGTGAAGGTCGGAGTCAAC |
|  | GGGGTCATTGATGGCAACAATA |
| *ASCL1* | CCCAAGCAAGTCAAGCGACA |
|  | AAGCCGCTGAAGTTGAGCC |
| *FOXA1* | GCAATACTCGCCTTACGGCT |
|  | TACACACCTTGGTAGTACGCC |
| *MYCN* | ACCCGGACGAAGATGACTTCT |
|  | CAGCTCGTTCTCAAGCAGCAT |
| *AURKA* | CAGACTGGATACCGGGACC |
|  | CTTCAGCACGTTTTTGCACTG |
| *BRN2* | CGGCGGATCAAACTGGGATTT |
|  | TTGCGCTGCGATCTTGTCTAT |
| *EZH2* | AATCAGAGTACATGCGACTGAGA |
|  | GCTGTATCCTTCGCTGTTTCC |
| *REST* | TCTCGGAGGTGGAGTACCTG |
|  | CCCAGTAAACGAGGTGACCAAA |
| *PEG10* | AGCAGTCGGAGGAGAACAAC |
|  | CACTGGGCCATGAAAGGAG |
| *SOX2* | CTCGTGCAGTTCTACTCGTCG |
|  | AGCTCTCGGTCAGGTCCTTT |
| *SOX9* | AGCGAACGCACATCAAGAC |
|  | CTGTAGGCGATCTGTTGGGG |
| *SOX11* | AGCAAGAAATGCGGCAAGC |
|  | ATCCAGAAACACGCACTTGAC |
| *ONECUT2* | GGAATCCAAAACCGTGGAGTAA |
|  | CTCTTTGCGTTTGCACGCTG |
| **CHIP-qPCR primer** | |
| *FOXA2* | AGGCTAACCCAGAACAGAAG |
|  | AGAGAAGAATCCAGGAATCA |
| Negative control | ATTCCCAATCTTGACACGG |
|  | GGTATCCCTCCCTCCTTCT |
